# No evidence of molecular markers of piperaquine resistance in southeastern Nigeria

**DOI:** 10.1186/s12936-025-05579-0

**Published:** 2025-09-23

**Authors:** Moses Ikegbunam, Vasileios Tzirtziganis, Miriam Rodi, Linda Anagu, Lais Pessanha de Carvalho, Juliana Inoue, Jana Held

**Affiliations:** 1 Department of Pharmaceutical Microbiology and Biotechnology, Nnamdi Azikiwe University, Awka, Nigeria; 2 Molecular Research Foundation for Students and Scientists, Nnamdi Azikiwe University, Awka, Nigeria; 3 Institute of Tropical Medicine, Eberhard Karls University, Tübingen, Germany; 4 Centre de Recherches Médicales de Lambaréné (CERMEL), Lambaréné, Gabon; 5 German Center for Infection Research (DZIF), Partner Site Tübingen, Tübingen, Germany

**Keywords:** Piperaquine, Resistance, *Plasmodium falciparum*, Southeastern Nigeria, *Pfcrt*, Plasmepsin 2, Plasmepsin 3, Nigeria

## Abstract

**Background:**

Artemisinin‑based combination therapy (ACT) remains the first‑line treatment for uncomplicated malaria, yet its long‑term efficacy is threatened by *Plasmodium falciparum* resistance to both artemisinin derivatives and partner drugs. Routine surveillance of clinical efficacy and molecular‑resistance markers is therefore essential. Piperaquine (PPQ), the partner drug in dihydroartemisinin–piperaquine (DHA‑PPQ) is already compromised in Southeast Asia, and key PPQ‑resistance mutations have begun to surface in parts of Africa. No such data are available from Nigeria. Three PPQ‑resistanceassociated singlenucleotide polymorphisms (SNPs)—T93S, H97Y and F145Ilocated in exons 2 and 3 of the *P. falciparum* chloroquineresistance transporter gene (*pfcrt*), and copy‑number variation of plasmepsin 2/3 (*pfpm2/3*), were retrospectively investigated using 299 archived patient isolates collected in Awka (n = 200) and Onitsha (n = 99), South‑East Nigeria (2018–2019).

**Methods:**

Allelic states at *pfcrt* codons 93, 97 and 145 were determined by nested PCR followed by Sanger sequencing. *Pfpm2/3* copy number was quantified by real‑time qPCR, using *β‑tubulin* as the single‑copy reference. All assays incorporated PPQ‑resistant RF7 and wild‑type 3D7 control strains, plus no‑template controls.

**Results:**

High‑quality amplification was achieved for 268 of 299 isolates (89.6%). None harboured the *pfcrt* T93S, H97Y or F145I mutations, and all exhibited single‑copy *pfpm2* and *pfpm3*. Two novel exon‑2 variants (K115I and synonymous L111L) were each detected once (0.4%) and are regarded as neutral polymorphisms without known phenotypic impact.

**Conclusions:**

The complete absence of validated PPQresistance markers indicates that piperaquine remains a viable partner drug in DHA‑PPQ for South‑East Nigeria. Nonetheless, because DHA‑PPQ is increasingly available over the counter, ongoing molecular and therapeutic‑efficacy surveillance is imperative to detect any future emergence of resistance.

**Supplementary Information:**

The online version contains supplementary material available at 10.1186/s12936-025-05579-0.

## Background

Artemisinin-based combination therapy (ACT) has been the first-line antimalarial treatment for uncomplicated falciparum malaria since 2005 in Nigeria due to emergence of chloroquine (CQ) resistance and increased failure rate of treatment with CQ [1]. The *Plasmodium falciparum* chloroquine resistance transporter (PfCRT) is largely responsible for this treatment failure and also contributes substantially to multidrug resistance. The substitution of lysine by threonine at codon 76 (K76T) in the *pfcrt* gene is the most important molecular marker to monitor CQ resistance in the field [2, 3]. CQ belongs to the 4-aminoquinoline group of antimalarials.

ACT is a combination of one rapidly acting artemisinin derivative that is readily eliminated and a long-acting partner drug. One of the partner drugs used in ACT is piperaquine (PPQ), a 4-aminoquinoline, partnered with dihydroartemisinin (DHA). PPQ, like other quinoline-based antimalarials, accumulates in the parasite’s digestive vacuole, where it binds to ferriprotoporphyrin IX (free haematin) and inhibits its detoxification into haemozoin. This leads to the accumulation of toxic free haematin, which disrupts parasite metabolism and ultimately results in parasite death [4].

However, the increasing resistance against both artemisinin and PPQ in Southeast Asia is leading to a significant increase in therapeutic failure in patients receiving DHA-PPQ treatment in this area [5]. Genome wide association studies have shown that PPQ resistance is strongly associated with the amplification of the aspartic proteinase genes plasmepsin 2 (*pfpm2*) and plasmepsin 3 (*pfpm3*) on chromosome 14 and with novel mutations in the *pfcrt* gene [6, 7]. Single nucleotide polymorphisms (SNPs) in *pfcrt* at positions downstream of the CQ resistance locus K76T, namely amino acids 72–76, T93S, H97Y, F145I, I218F, M343L, and G353V together with an amplified *pfpm2/3* majorly mediate PPQ resistance as demonstrated in recent studies [6, 8]. More recently, an insertion/duplication at codon 350 of *pfcrt* (designated crt 350X) has emerged as an additional, high‑level resistance factor: although still undetected in 602 African isolates [9], crt 350X is strongly associated with DHA‑PPQ treatment failure in the Guiana Shield [10] and is increasingly prevalent in South American foci under sustained PPQ pressure [11].

Here, we assess whether these molecular markers associated with PPQ resistancespecifically, the SNPs T93S, H97Y, and F145I mutations in exons 2 and 3 of *pfcrt* and the increased copy number of the aspartic proteinase genes *pfpm2* and *pfpm3* are circulating in Nnewi Town, Southeast Nigeria, as these mutations have shown strong associations with PPQ resistance in previous studies [7]. Even though the first line therapy for uncomplicated malaria in Nigeria is artemether-lumefantrine and artesunate-amodiaquine, the combination of DHA-PPQ is nevertheless readily available on the private market with little regulatory control. The formal addition of PPQ to combination therapy is currently being considered in Nigeria, emphasizing the need to monitor resistance markers [12]. Early detection of drug-resistant parasites is crucial for timely intervention. Notably, no studies in Nigeria have investigated these specific PPQ resistance markers, making this the first effort to assess their presence in the region.

## Methods

### Study area and design

This study analysed 299 *P. falciparum* positive DNA samples collected in two cross‑sectional surveys conducted at local hospitals in Awka, September–December 2019 (n = 200) and Onitsha, February–March 2018 (n = 99). A first publication analysed the samples for the prevalence of *P. falciparum* histidine-rich protein 2 and 3 gene deletions [13]. DNA was extracted within 48 h after blood draw and archived in – 20 ºC freezer before downstream assays.

### Parasite strains and clinical samples

*Plasmodium falciparum* 3D7 (CQ-sensitive, obtained from BEI Resources; reference number MRA-102) and RF7 (provided by Dr. David Fidock, Columbia University, NY) were used in this study. RF7 is PPQ- and DHA-resistant Cambodian clinical isolate that naturally expresses either the K13 C580Y mutation or the PfCRT Dd2 + M343L isoform [14] with pm2/3 multicopies [7]. The parasites were maintained in O + erythrocytes at 2.5% haematocrit in RPMI 1640 medium containing 50 µg/ml gentamicin (Gibco), 25 mM HEPES (Sigma-Aldrich), 2.1 mM l-Glutamine (Gibco), and 0.5% (wt/vol) AlbuMAX II (Life technologies) containing 73 µM hypoxanthine and 1.9 mM sodium bicarbonate [15]. *Plasmodium falciparum*–positive blood samples were collected during two hospital‑based cross‑sectional surveys in Anambra State, south‑east Nigeria: Chukwuemeka Odimegwu Ojukwu Teaching Hospital, Awka (September–December 2019; n = 200) and General Hospital in Onitsha (February–March 2018; n = 99), as mentioned previously [13]. Consecutive patients presenting with uncomplicated malariadefined as an axillary temperature ≥ 37.5 °C or a history of fever plus at least one additional symptom (headache, vomiting, or malaise)were invited to participate after written informed consent (guardian consent for minors). In total 299 participants were enrolled (mean age 16 years; 135 males and 164 females). Genomic DNA extracted from these bloods yielded successful *pfcrt* exon‑2 and 3 amplification in 268 samples (89.6%), which constitute the dataset analysed in the present study*.*

### DNA extraction and amplification of exon 2 and 3 of *pfcrt*

DNA was extracted using QIAamp DNA Blood Mini Kit (250) (Qiagen). DNA yield was quantified using a Nanospec 1000 (Thermo Fisher Scientific USA). Amplification of exon 2 of *pfcrt*, containing T93S and H97Y loci, and exon 3 of *pfcrt*, containing F145I locus, was carried out by PCR in a Master Cycler Nexus Gradient (Eppendorf, US) in 25 μl reaction mixture containing 2.5 μl of 10 × Coral Load PCR buffer, 0.8 μl of 25 mM MgCl2, 0.5 μl of each of the 10 mM forward and reverse primer, 0.5 μl of 10 mM dNTPs, 0.2 μl Taq DNA Polymerase (5 units/μL) and 19 μl of nuclease free water. The primers used were published in a previous study [16] (Additional file 1). Cycling conditions were initial denaturation at 95 °C for 5 min, followed by 40 cycles of denaturation at 95 °C for 30 s, annealing at 53 °C for 45 s and elongation at 60 °C for 45 s. A final elongation was done at 60 °C for 10 min.

### Amplicon detection

PCR products were separated directly on a 1.5% unstained agarose gel (SeaKem L). 2.5 µL of each DNA sample were mixed with 2.5 µL of SYBR Green loading buffer (to produce 150 µL SYBR loading buffer: 100 µL H2O was combined with 50 µL SYBR Green) and then loaded onto wells of the gel. The gel was run at 120 V for 60 min and PCR products were visualized by UV illumination and photographed using INTAS GelStick IMAGER UV-Transilluminator.

### Sequencing and alignment of the exons 2 and 3 of *pfcrt* gene

All PCR products to be sequenced were purified using Exo-SAP-IT (USB, Affymetrix, USA). Subsequently, 1 µl of the purified product was used as a template for direct sequencing with forward and reverse primers using the Big Dye terminator v. 2.0 cycle sequencer, according to the manufacturer's instructions. Sequence reads were 150 bp for exon 2 and 110 bp for exon 3 for all the samples. The sequencing reads were then analysed, interpreted, and aligned to the exons 2 and 3 of *pfcrt* from *P. falciparum* 3D7 reference genome (PF3D7_0709000) using Geneious Sequence Alignment Editor 7.7.1.0.

### Copy number variation analysis for plasmepsin 2 and 3

For all 268/299 DNA samples, copy number variation of *pfpm2* (PF3D7_1408000) and *pfpm3* (PF3D7_1408100) was measured by duplex qPCR assays targeting either *pfpm2* or *pfpm3* and *pfβ-tubulin* in the Light Cycler 480 II/1 (Roche) using SensiFAST™ Probe No-ROX (Meridian Biosciences), 2 µl of DNA sample, 300 nM primers and 100 nM probes in a 10 µl reaction volume. Cycling conditions were initial denaturation at 95 °C for 10 min, followed by 50 cycles of denaturation at 95 °C for 15 s, annealing and elongation at 58 °C for 60 s. The single copy β-*tubuli*n (PF3D7_1008700) gene was used as a reference gene. The primers to the target genes, probes, protocols, were used as published [17]. All primers were manufactured, and HPSF/Salt®-purified by Integrated DNA Technologies ®. The relative copy number (RCN) of each target gene in each sample was calculated based on the 2 − ΔΔCt method for relative quantification. ΔΔCt was calculated as (Ct *pfpm2/3*(sample) − Ct *β-tubulin* (sample)) − (Ct *pfpm2/3* cal − Ct *β-tubulin* cal), where cal is the calibration control of genomic 3D7 DNA with one copy of both *β-tubulin* and *pfpm2* and *pfpm3* [17]. Standard curves using 3D7 strain were used to check the amplification efficiency and it ranged from 98 to 100%. DNA from the RF7 *P. falciparum* strain with two copies of *pfpm2/3* was used as positive control. Ct values were calculated with the second derivative maximum method using the Light Cycler 480 soft-ware (v. 5.1.1). The qPCR analysis of the samples was done in duplicates. A *pfpm2* or *pfpm3* RCN > 1.5 was defined as an amplification of the gene. The mean RCN for all samples presented in the copy number graphs are derived from raw data.

### Analysis

GraphPad Prism 8 was used to create all the figures associated with the analysis of the results.

## Results

### *Pfcrt T93S, H97Y, F145I* SNPs are absent in clinical isolates from Anambra State

268 patient samples were successfully amplified and sequenced. None of these samples had any of the three different SNPs, T93S and H97Y in exon 2, and F145I in exon 3, that have been associated with PPQ resistance. Other non-synonymous and synonymous mutations present in exon 2 were found in 2 of the 268 patient samples. These mutations were confirmed by a second independent PCR and sequencing run and are shown in Table 1.

**Table 1:** Novel mutations found in exon 2 of *pfcrt* from clinical samples from Anambra State, Southeast Nigeria

Sample	Codon mutation	Amino acid change	Haplotype
C60	115: AAA → ATA	K → I^a^	K115I
M39	111: TTA → TTG	L → L	L111L

^a^ A non-synonymous mutation. Numbers are the position of the amino acid where the mutation occurs

### Copy number variation of pfpm2 and pfpm 3

**Fig. 1:**
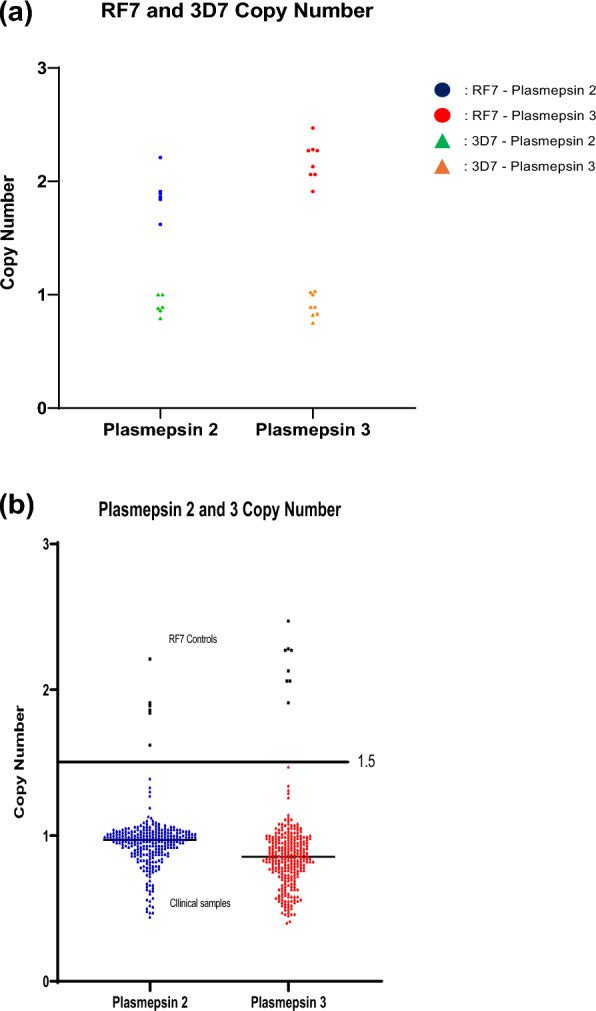
**A** *Plasmepsin 2, plasmepsin* 3 relative copy numbers validation for the RF7 and 3D7 strains. The median *pfpm2* copy number was 2.02 and for the *pfpm3* gene 2.27 in the RF7 strain. A relative copy number of > 1.5 was defined as gene amplification. **B** Relative copy number of *pfpm2* and *pfpm3* in the clinical samples from patients visiting Nnamdi Azikiwe University Teaching Hospital in Nnewi Town, Anambra State, Southeast Nigeria. *pfpm2* and *pfpm3* copy numbers for the 268 clinical samples (in blue and red) and the RF7 strain (in grey). None of the clinical samples showed a gene amplification (relative copy number of > 1.5)

Copy numbers of *pfpm2* and *pfpm3* were analysed as potential markers of PPQ resistance. For validation of amplification, the *P. falciparum* RF7 strain was used and the amplification is shown in Fig. 1A. None of the clinical samples had a multicopy of either the *pfpm2* or *pfpm3* gene, Fig. 1B. The copy number of *pfpm2* and *pfpm3* in all tested clinical samples was below 1.5 copies. The median copy number of *pfpm2* was 0.94 while that of *pfpm3* was 0.84.

## Discussion

The burden of malaria in Africa, and especially in Nigeria remains high. In 2023, the death toll of malaria worldwide was 597,000 with 73.7% being children [18]. Nigeria accounted for 26% of cases on the African continent [18]. The progress with high impact strategies especially in Nigeria remains barely visible and the development of resistance to the currently available antimalarials remains a threat to public health. PPQ was initially utilized as an alternative to CQ, as the first-line therapy for CQ-resistant *P. falciparum* in the 1970 s, due to the fact it was considered at least as effective and better tolerated [19] and has been used for a long time in China [20]. Due to the development of PPQ-resistant strains of *P. falciparum* and the appearance of the artemisinin derivatives, the use of PPQ as monotherapy diminished in the 80 s [21]. PPQ resurfaced in the 90 s as a partner drug for ACT [4]. In this current study, there was no evidence that PPQ resistant markers are present in parasites circulating in the country, as none of the investigated parasites in the 268 samples had multiple copies of *pfpm2* and/or *pfpm3.*

The investigated mutations in *pfcrt*, T93S, H97Y, and F145I, which have been associated with treatment failures with PPQ, were also not found in the present study. Such mutations are frequently detected in the Greater Mekong Subregion (GMS), but less prevalent or almost non-existent in Thai-Myanmar border region [22] and northwestern Thailand [16]. We did not sequence the exo-E415G mutation, linked to PPQ resistance in Cambodia [6], but future research should include it in broader molecular surveillance efforts due to its relevance in parts of Southeast Asia. Two novel exon‑2 variants, K115I and the synonymous L111L, were each observed in a single isolate (0.4%) and are considered neutral, polymorphisms with no known association to antimalarial resistance.

It has been demonstrated that amplification of *pfpm2* is not restricted to the eastern GMS [23]. Hence, amplification of *pfmp2* has also been reported in some parts of Africa including Tanzania (10%) [24], Mali (11%) [25], Uganda (33.9%) [26], Mozambique (12.5%) [26], Burkina Faso (30.5%) [26], Gabon (11.3%) [26], and also in one case in two investigated samples from the Democratic Republic of Congo (50%) [26]. Nevertheless, DHA + PPQ remains effective in African countries and *pfpm2* amplification was not associated with DHA + PPQ treatment failure in patients from Ethiopia [27] and Cameroon [28]. These reports raise the question whether other mutations could be involved in PPQ resistance in African strains, differing from the main pattern observed in Southeast Asia. In this way, research on molecular markers associated with antimalarial resistance in the African parasite population could be expanded [26, 28]. Although amplification of *pfpm2* has been shown not to be relevant to the development of PPQ resistance [7], recent studies have shown that epistasis between the amplified *pfpm2/3* and certain mutations downstream of the 72–76 *pfcrt* 4-aminoquinoline resistance locus in *Plasmodium* might enhance the generation of high-level PPQ resistance, although this synergy decreases the ability of such mutants to survive [29]. One of the limitations of the current study is that the molecular markers were not assessed along with treatment outcomes, which would provide a more comprehensive understanding.

This study did not detect any copy number increase in the markers related to PPQ resistance in samples from southeastern Nigeria. In general, there are no indications that current artemisinin-based combinations have lost efficacy in Nigeria, and drug resistance is not the major driver of the country`s high malaria burden. However, the absence of PPQ resistance markers does not eliminate the risk of emerging resistance. Continuous monitoring and surveillance remain essential to detect resistance early and ensure the long-term effectiveness of malaria treatments.

### Limitations

This study provides only a retrospective, molecular snapshot of piperaquine‑resistance markers in Anambra State. First, the analysis relied on archived blood‑spot DNA collected in 2018–2019, so the findings may not reflect current parasite populations or emergent mutations since DHA‑PPQ use has expanded. Second, no phenotypic data—ex‑vivo PPQ IC₅₀s, piperaquine survival assays, or day‑42 therapeutic‑efficacy outcomeswere available to confirm the clinical relevance of the genotypes observed. Third, only the three validated *pfcrt* exon‑3 mutations (T93S, H97Y, F145I) plus *pfpm2/3* copy number were screened; other loci of interestincluding *pfcrt* K76T, the exo‑E415G mutation, and the insertion/duplication at codon 350 (crt 350X)were not sequenced due to DNA‑quantity and budget constraints and will require targeted amplicon or whole‑genome sequencing in future surveys. Fourth, sampling was confined to two semi‑urban hospitals in a single state, potentially limiting geographic representativeness, and the moderate sample size (268 successfully genotyped isolates) reduces power to detect very low‑frequency variants.

## Supplementary Information

Additional file1

## Data Availability

Other supporting data like agarose gels images, sequence alignments and raw Ct values can be provided by the authors upon request.
