# Supplementary material for: No evidence of molecular markers of piperaquine resistance in southeastern Nigeria
Source: Malar J. 2025 Sep 23;24:287. doi: 10.1186/s12936-025-05579-0 (PMC12455774; doi:10.1186/s12936-025-05579-0)
Supplement: Supplementary file 1 — Additional file1 [file 12936_2025_5579_MOESM1_ESM.pdf]

Supplementary File:

Table 1. *Primers used for analysis of pfcrt markers associated with DHA-PPQ resistance.*

NAME	5´ - 3´ SEQUENCE
***pfcrt* exon 2 forward**	-TTAAGTATTATTTATTTAAGTGTATGTG-
***pfcrt* exon 2 reverse**	-GATTTATCTTACTTTTGAATTTCCC-
***pfcrt* exon 3 forward**	-GACACCGAAGCTTTAATTTAC-
***pfcrt* exon 3 reverse**	-GAACATATTAATAGGAATACTTAATTG-

Table 2. *List of primers for the qPCR of β-tubulin, plasmepsin 2 and plasmepsin 3*

PCR	PRIMER NAME	SEQUENCES
β-tub	Pfβ-tubulin_CN_F	5′-TGATGTGCGCAAGTGATCC-3′
	Pfβ-tubulin_CN_R	5′-TCCTTTGTGGACATTCTTCCTC-3′
pfpm2	PfPM2_CN_F	5′-ATGGTGATGCAGAAGTTGGA-3′
	PfPM2_CN_R	5′-AACATCCTGCAGTTGTACATTTAAC-3′
pfpm3	PfPM3_CN_F	5′-CCACTTGTGGTAACACGAAATTA-3′
	PfPM3_CN _R	5′-TGGTTCAAGGTATTGTTTAGGTTC-3′
